# Knowledge and Attitude of Emergency Nurses Regarding Work‐Related Legal Issues in Erbil Hospitals: A Cross‐Sectional Study, 2023–2024

**DOI:** 10.1002/hsr2.70496

**Published:** 2025-03-02

**Authors:** Hardi Abdulqadir Hasan, Abdulmalik Fareeq Saber, Alireza Nikbakht Nasrabadi

**Affiliations:** ^1^ Department of Medical‐Surgical School of Nursing and Midwifery Tehran University of Medical Sciences Tehran Iran; ^2^ Department of Psychiatric Nursing, School of Nursing & Midwifery Tehran University of Medical Sciences Tehran Iran; ^3^ Psychiatric and Mental Health Nursing, College of Nursing Hawler Medical University Erbil Iraq

**Keywords:** attitudes, emergency nursing, health knowledge, health policy, legal knowledge, risk management

## Abstract

**Background and Aim:**

In Erbil, Iraq, emergency nurses frequently encounter complex legal issues in their professional practice, impacting their knowledge, attitudes, and interference with legal matters. This study aimed to analyze the level of legal knowledge, attitudes toward legal issues, and the extent of legal interference among emergency nurses in Erbil.

**Methods:**

This cross‐sectional study was conducted from January 1 to February 5, 2024, in eight major public hospitals in Erbil. Purposive sampling was used to collect data using a comprehensive questionnaire. The questionnaire included demographic information and the Emergency Nursing Legal Issues Assessment Scale (ENLIAS), which measured legal knowledge, attitudes toward legal issues, and interference with legal matters. Statistical analysis was performed using Stata version 12 (StataCorp LLC, College Station, TX). Pearson correlation analyses and multiple linear regression were conducted to assess the correlations between legal knowledge, attitudes, interference with legal issues, and demographic variables.

**Results:**

A total of 254 emergency nurses participated in the study. The mean score for legal knowledge was 6.00 ± 3.25, indicating a moderate level of knowledge. The mean score for attitudes toward legal issues was 5.35 ± 2.08, also reflecting a moderate level. The mean score for interference with legal issues was 24.69 ± 5.16, indicating medium interference. There was a significant positive correlation between legal knowledge and good attitude (*r* = 0.47, *p *< 0.001), suggesting that higher legal knowledge is associated with a more positive attitude toward legal issues. Conversely, there was a significant negative correlation between legal knowledge and interference with legal issues (*r* = −0.55, *p* < 0.001), indicating that greater legal knowledge is associated with lower interference in legal matters.

**Conclusions:**

The study demonstrated that emergency nurses in Erbil have moderate levels of legal knowledge and attitudes toward legal issues, with medium levels of interference in legal matters. Policymakers and healthcare providers should develop targeted educational interventions to enhance legal literacy and support nurses in effectively managing legal issues.

## 1 Introduction

Work‐related legal issues are a pressing concern in the healthcare sector, especially for emergency nurses who frequently encounter complex and high‐stakes situations [1, 2]. In Erbil, the capital city of the Kurdistan region in Iraq, a thorough understanding of these legal issues is crucial due to the unique healthcare challenges and ever‐changing legal landscape. Emergency nurses in this region are at the forefront of providing immediate care, which requires them to have a comprehensive grasp of their legal responsibilities and the potential implications. While legal issues are relevant to all nurses, emergency department nurses face heightened challenges due to the urgent and unpredictable nature of their work. Their responsibilities often involve split‐second decision‐making and high‐stakes situations, making them more susceptible to legal risks compared to nurses in other specialties. Moreover, the healthcare system in Erbil is still in the early stages of development, characterized by limited resources, inconsistent training opportunities, and evolving healthcare policies. This creates unique challenges for emergency nurses, who should navigate legal and clinical complexities without the robust support systems available in more developed regions. Globally, approximately 10% of nurses face legal issues annually, often related to malpractice and workplace regulations [3]. In the UK, around 12% of nurses report being involved in legal disputes within their careers [4]. These figures highlight the pervasive nature of legal concerns in the healthcare sector worldwide. In Iraq, current statistics on legal issues among healthcare workers, including nurses, are not available. In Erbil specifically, there is also a lack of recent data on the prevalence of legal challenges faced by emergency nurses, underscoring the importance of legal literacy among healthcare providers in this region.

This study is significant because of the high‐pressure environment in emergency departments, where nurses should make swift decisions that can have significant legal consequences. Legal knowledge is particularly vital in areas such as consent, patient confidentiality, and handling of medical records [5]. Previous research have indicated that a lack of legal knowledge among healthcare professionals can result in legal violations and subsequent litigation, underscoring the importance of this study [6, 7]. Additionally, understanding the attitudes of nurses toward these legal issues can provide insights into their preparedness and confidence in navigating legal situations [8]. This is especially critical in Erbil, where the healthcare infrastructure is still developing and the legal framework governing healthcare practices is continually evolving.

Existing research on this topic has mainly focused on developed countries, with little attention given to regions like Erbil, where the healthcare system is still in its early stages of development. Studies have shown that nurses' knowledge and attitudes toward legal issues vary significantly based on geographic location, educational background, and access to continuous professional development [9, 10]. For instance, nurses in countries with robust legal frameworks and ongoing legal education programs tend to possess a better understanding and more positive attitudes toward legal issues compared to those in regions with limited resources [11]. Furthermore, the absence of standardized legal training for nurses in Erbil highlights the necessity of this study. While some nurses may have received formal education on legal issues, others may rely solely on experience or sporadic training sessions, creating a disparity in knowledge and attitudes.

The relationship between nurses' knowledge and their attitudes toward legal issues is an important area of investigation. Previous studies indicate that increased knowledge often corresponds with more positive attitudes and greater confidence in managing legal matters [6, 12, 13]. However, the direction of this relationship and its consistency across different healthcare settings have not been thoroughly explored. Despite existing research, several gaps remain. Most studies have focused on high‐income countries, leaving a significant gap in our understanding of dynamics in lower‐middle‐income regions. Additionally, the influence of local legal frameworks and cultural factors on nurses' legal knowledge and attitudes has not been extensively studied. Addressing these gaps is crucial for developing targeted interventions that can enhance legal literacy among nurses, ultimately improving patient care and reducing legal risks for healthcare providers. Therefore, the main aim of this study is to analyze the level of knowledge, attitudes, and the extent of nurses' involvement in legal issues in the context of emergency nursing in Erbil.

### 1.1 Research Question

What are the levels of knowledge, attitudes, and legal interference among emergency nurses in Erbil hospitals, and how are these factors related to each other?

## 2 Methods

### 2.1 Study Design, Setting, Period, and Sampling

This study was a cross‐sectional study conducted in Erbil, Iraq, involving eight major public hospitals. The purposive sampling method was used to collect data from January 1 to February 5, 2024. All governmental hospitals with emergency departments in Erbil were chosen to ensure comprehensive representation of emergency nursing practices in the region. The selected hospitals were Nanakaly Hospital for Hematology & Oncology, Rozhawa Emergency Hospital‐Erbil, Rojhalat Emergency Hospital, Central Emergency Hospital, EMC‐Emergency Hospital, Maternity Teaching Hospital, Raparin Teaching Hospital for Children, and Hawler Psychiatric Hospital.

### 2.2 Sample Size

The sample size for this study was determined based on the total number of emergency nurses available in the eight selected hospitals in Erbil. There were 254 nurses in these hospitals, and we collected data based on the availability of the nurses. We were able to include all 254 available nurses in our study.

### 2.3 Inclusion/Exclusion

The inclusion criteria for participants included nurses of both genders, with nursing qualifications at the preparatory, diploma, or bachelor's level. Additionally, they needed to be currently working in the emergency department as nursing staff, and have at least 6 months of recent experience in this role. On the other hand, nurses who declined to participate in the study or were absent from work for any reason during the data collection period were excluded from the study.

### 2.4 Study Tools and Data Collection

The questionnaire was divided into two main parts. The first part gathered demographic data, including Date of Birth, Gender, Highest Educational Qualification, Current Employment Status, Type of Hospital, and Number of Years Working in Healthcare. The second part was a self‐structured questionnaire, the Emergency Nursing Legal Issues Assessment Scale (ENLIAS), which contained 50 questions designed to evaluate three distinct dimensions: the level of knowledge, attitude toward legal issues, and the extent of interference with legal matters among emergency nurses. The questionnaire was provided in English, as the nurses' education was conducted in English, and any unclear questions were explained by the researchers. Data were collected by distributing questionnaires to participants who met the inclusion criteria. Each participant was allotted a total of 10–15 min to complete the questionnaire.

### 2.5 Pilot Study

The study questionnaire, called the ENLIAS, was initially tested with a group of 25 nurses who had previous experience working in emergency departments. The testing took place between October 2 and November 2, 2023, and aimed to assess the internal consistency and reliability of the questionnaire items before using them in the actual study. The internal consistency of the items was calculated using Cronbach's alpha [14]. The overall Cronbach's alpha was calculated as 0.91, indicating an excellent level of internal consistency and reliability. It is important to note that the data from this initial study were excluded from the final analysis.

### 2.6 Measures

#### 2.6.1 Sociodemographic Characteristics

The first section of the questionnaire included sociodemographic information of the emergency nurses, such as date of birth, gender, highest educational qualification, current employment status, type of hospital, and number of years working in healthcare.

#### 2.6.2 ENLIAS

To assess work‐related legal issues among emergency nurses in selected hospitals of Erbil, we used the ENLIAS. This questionnaire, consisting of 50 questions, was carefully designed to evaluate three dimensions: level of knowledge, attitude toward legal issues, and extent of interference with legal matters among emergency nurses. Due to the lack of existing tools specifically designed to assess legal issues in emergency nursing, we developed the ENLIAS based on expert opinions from nursing professionals, legal advisors, and academics familiar with emergency healthcare settings. To quantitatively measure nurses' level of knowledge of legal issues, specific questions (2, 4, 5, 7, 12, 14, 15, 23, 24, 26, 32, 34, 36, 38, and 46) were aggregated. The knowledge scale, containing 15 items, was categorized as follows: scores of 1‐5 indicated low knowledge, 6–10 indicated medium knowledge, and 11–15 indicated high knowledge. These score ranges were established to identify areas requiring educational intervention: low scores indicate insufficient knowledge that may lead to legal risks, while higher scores demonstrate preparedness and understanding of legal responsibilities. Similarly, the assessment of attitudes was based on the aggregation of responses to questions 2, 7, 10, 17, 21, 30, 33, 35, 44, and 50. The attitude scale, containing 10 items, was categorized as follows: scores of 1‐3 indicated low attitude, 4–6 indicated medium attitude, and 7–10 indicated high attitude. Higher attitude scores represent greater positivity and confidence in addressing legal issues, while lower scores may indicate hesitancy or lack of preparedness. The degree of interference with legal issues was categorized as low (0%–16.5%), medium (17%–33%), and high (33.5%–50%). This categorization was determined by summing up responses to all questions, with each “Yes“ answer scoring 1 point, indicating acknowledgment of legal concerns. “No” responses generally scored 0 points, indicating the absence of such concerns. However, for specific questions (2, 4, 5, 7, 8, 10, 12, 14, 15, 19, 23, 24, 26, 28, 32, 34, 36, 37, 38, 42, 43, 46, 48, 50), a “No” response was assigned 1 point, suggesting that a negative response in these instances indicated interference with legal issues.

### 2.7 Ethical Approval and Informed Consent

This study followed the Institutional Research Ethics Board and the Declaration of Helsinki guidelines. Ethical approval for the study was obtained from the School of Nursing and Midwifery & Rehabilitations Ethics Committee of Tehran University of Medical Sciences, with the code IR.TUMS. FNM.REC.1402.185 granted on December 16, 2023. Oral informed consent was obtained from all participants before they filled out the questionnaires.

### 2.8 Statistical Analysis

Data were summarized and reported with frequency and percentage for qualitative variables. Quantitative variables were presented with mean and standard deviations. The data were weighted to the population and standardized according to WHO population estimates for 2000–2025 using survey analysis. The relationships between nurses' knowledge, attitudes, and involvement in legal issues were assessed using Pearson's correlation coefficient. The adjusted association between these variables and other confounding factors was evaluated using multiple linear regression analysis. Data analysis was performed using Stata version 12 (StataCorp LLC, College Station, TX), with significance levels considered at *p* < 0.001.

## 3 Results

### 3.1 Demographic and Clinical Characteristics

A total of 254 responses were analyzed. The ages of participants ranged between 21 and 60 years, with a mean age of 36.98 ± 7.99 years. The gender distribution showed that 54.3% (138) were male and 45.7% (116) were female. Marital status indicated that 74.4% (189) of participants were married, 21.7% (55) were single, and 3.9% (10) were divorced. In terms of educational qualifications, 51.6% (131) held a Bachelor's degree, 11.4% (29) had a high school diploma, and 33.5% (85) had other qualifications. Employment status showed that 70.5% (179) were employed full‐time, while 15.4% (39) were part‐time, and 12.2% (31) were not employed. Regarding emergency department experience, 32.3% (82) had more than 10 years of experience, while 29.1% (74) had 6 months to 1 year. The majority of nurses had moderate legal knowledge (50.0%, 127; mean score: 6.00 ± 3.25) and moderate attitudes toward legal issues (53.1%, 135; mean score: 5.35 ± 2.08). In terms of legal issues interference, 91.7% (233) experienced medium interference, with a mean score of 24.69 ± 5.16. Detailed demographics and other variables are presented in Table 1.

**Table 1:** Demographic and clinical characteristics of emergency nurses.

Variables	Characteristics *n* = 254	*F*	%
Age (year)	21–30	53	20.87
	31–40	128	50.39
	41–50	55	21.65
	51–60	18	7.09
	Mean ± SD	36.98 ± 7.99	
Gender	Male	138	54.3
	Female	116	45.7
Marital status	Single	55	21.7
	Married	189	74.4
	Divorced	10	3.9
Highest educational qualifications	High school diploma	29	11.4
	Bachelor's degree	131	51.6
	Master's degree	7	2.8
	Doctorate	2	0.8
	Others	85	33.5
Current employment status	Full‐time	179	70.5
	Part‐time	39	15.4
	Not employed	31	12.2
	Student	5	2.0
Nurses' experiences in the emergency department	6 months–1 year	74	29.1
	1–5 years	63	24.8
	5–10 years	35	13.8
	More than 10 years	82	32.3
Experiences of nurses in other departments	Less than 1 year	49	18.3
	1–5 years	76	29.9
	5–10 years	48	18.9
	More than 10 years	81	32.9
Knowledge levels	Poor	108	42.5
	Moderate	127	50.0
	Good	19	7.5
	Mean ± SD	6.00 ± 3.25	
Attitude levels	Poor	50	19.7
	Moderate	135	53.1
	Good	69	27.2
	Mean ± SD	5.35 ± 2.08	
Legal issues interference levels	Low legal issues interference	14	5.5
	Medium legal issues interference	233	91.7
	High legal issues interference	7	2.8
	Mean ± SD	24.69 ± 5.16	

*Note: F* = frequency, % = percentage; knowledge was scored as low (1–5), medium (6–10), and high (11–15); attitude was scored as low (1–3), medium (4–6), and high (7–10); legal interference was categorized as low (0%–16.5%), medium (17%–33%), and high (33.5%–50%).

### 3.2 Correlation Between Legal Knowledge, Attitude, and Interference of Nurses to Legal Issues

The study examined the relationships between nurses' legal knowledge, attitudes toward legal issues, and their interference with legal matters. There was a significant positive correlation between legal knowledge and good attitude (*r* = 0.47, *p* < 0.001), indicating that higher legal knowledge is associated with a more positive attitude toward legal issues. Conversely, there was a significant negative correlation between legal knowledge and interference with legal issues (*r* = −0.55, *p* < 0.001), suggesting that higher legal knowledge is associated with less interference in legal matters. The correlation between good attitude and interference with legal issues was weak and not statistically significant (*r* = ‐0.07, *p* = 0.30). For further details, see Table 2.

**Table 2:** Correlation between legal knowledge, attitude toward legal issues, and interference of nurses to legal issues among emergency nurses (*n* = 254).

Variables	Pearson correlation	Legal knowledge	Good attitude	Interference of nurses to legal issues
Legal knowledge	Correlation coefficient	1.00	0.47**	−0.55**
	Sig. (2‐tailed)	—	*p* < 0.001	*p* < 0.001
	*N*	254	254	254
Good attitude	Correlation Ccoefficient	0.47**	1.00	−0.07**
	Sig. (2‐tailed)	*p* < 0.001	—	0.30
	*N*	254	254	254
Interference of nurses to legal issues	Correlation coefficient	−0.55**	−0.07**	1.00
	Sig. (2‐tailed)	*p* < 0.001	0.30	—
	*N*	254	254	254

**Correlation is significant at the 0.01 level (2‐tailed).

### 3.3 Correlation Between Legal Knowledge, Good Attitude, Interference of Nurses to Legal Issues, and Demographic Variables

The study also explored the relationship between legal knowledge, good attitude, and interference of nurses with legal issues, and demographic variables. Notably, gender showed a significant standardized coefficient (*B* = −0.12, *p* = 0.01), indicating that differences in gender are associated with variations in legal knowledge. Specifically, female nurses had higher legal knowledge scores compared to male nurses. Legal knowledge showed a significant positive standardized coefficient with good attitude toward legal issues (*B* = 0.45, *p* < 0.001), suggesting that a more positive attitude is strongly associated with higher legal knowledge. Conversely, legal knowledge had a significant negative standardized coefficient with interference of nurses with legal issues (B = −0.50, *p* < 0.001), indicating that greater knowledge is associated with lower interference in legal matters. For more details, refer to Table 3.

**Table 3:** Final model of multiple linear regression for assessing the association between legal knowledge, attitudes toward legal issues, interference of nurses to legal issues, and demographic variables among emergency nurses.

Variables	Coefficient standardized (B)	Coefficient Unstandardized (B)	95% Confidence Interval		*p*‐value*
			Lower	Upper	
Age	−0.02	−0.01	−0.06	0.04	0.70
Gender	−0.12	−0.77	−1.37	−0.17	0.01
Marital status	0.02	0.12	−0.55	0.79	0.72
Highest educational qualification	−0.05	−0.10	−0.30	0.09	0.29
Current employment status	0.05	0.22	−0.22	0.67	0.33
Nurses' experiences in ED	0.04	0.09	−0.17	0.36	0.49
Experiences of nurses in other departments	−0.10	−0.29	−0.59	0.01	0.06
Good attitude	0.45	0.70	0.56	0.85	0.001*
Interference of nurses to legal issues	−0.50	−0.32	−0.37	−0.26	0.001*

*Note:* Legal Knowledge is the dependent variable, Significance was set at *p* < 0.001. Those marked (*) specifically highlight significance at *p* < .001.

## 4 Discussion

Emergency nursing is a field that deals with complex and high‐stakes situations, often involving urgent care and critical decision‐making [15, 16]. In Erbil, the legal landscape is constantly changing and the healthcare challenges faced are unique, making it essential for emergency nurses to have a strong understanding of legal matters. Unfortunately, there is a lack of data on the legal awareness of emergency nurses in this region. Given the importance of legal sknowledge in nursing practice, we have undertaken this study to examine the level of knowledge, attitudes, and the extent to which nurses are involved in legal issues in the context of emergency nursing in Erbil.

The present study was conducted to analyze the level of knowledge, attitudes, and the extent of nurses' involvement in legal issues in the context of emergency nursing in Erbil. Overall, the results revealed that the participants had a moderate level of legal knowledge, a neutral to moderate attitude toward legal issues, and a relatively moderate level of interference with legal matters.

The demographic profile of our study participants, predominantly full‐time employed emergency nurses with varying educational qualifications and extensive experience, offers a comprehensive representation of the emergency nursing workforce in Erbil. The majority of nurses having over 10 years of experience in the emergency department aligns with global trends in nursing workforce demographics [17, 18]. This experienced cohort provides a solid foundation for understanding the long‐term implications of legal knowledge and attitudes in emergency nursing practice.

Our study found that emergency nurses in Erbil have moderate levels of legal knowledge and attitudes toward legal issues. This finding is consistent with previous research conducted in various countries, which consistently reported moderate to low levels of legal knowledge among nurses [19, 20]. The similarity in results across different healthcare systems suggests that inadequate legal education in nursing curricula may be a widespread problem. However, the specific context of emergency nursing in Iraq, with its unique challenges and legal framework, may contribute to the specific knowledge gaps observed in our study. The moderate level of knowledge indicates a need for targeted educational interventions to enhance legal literacy among emergency nurses in Erbil. A significant positive relationship was found between nurses' legal knowledge and their attitudes toward legal issues, which is an important finding from our study. This correlation suggests that as nurses become more informed about legal matters, they develop more positive attitudes toward addressing and managing legal issues in their practice. Similar associations have been reported in other studies, such as [21, 22]. The consistency of this relationship across different healthcare systems highlights the importance of legal education in shaping nurses' perceptions and approaches to legal matters. However, the strength of this correlation may vary depending on cultural and systemic factors, emphasizing the need for context‐specific educational strategies.

Interestingly, our study revealed a significant negative relationship between legal knowledge and interference with legal issues. This finding suggests that nurses with greater legal knowledge experience less interference or difficulty in managing legal matters in their professional roles. This inverse correlation aligns with research from other healthcare settings, which have demonstrated that improved legal literacy leads to better decision‐making and reduced legal complications [23, 24]. The similarity in findings across different contexts reinforces the protective role of legal knowledge in nursing practice. However, the specific factors contributing to reduced interference may vary between healthcare systems, warranting further investigation into the mechanisms by which legal knowledge mitigates challenges in the Iraqi emergency nursing context.

The gender differences observed in our study, with female nurses demonstrating higher levels of legal knowledge compared to their male counterparts, present an intriguing finding that contrasts with some previous research. Studies in other countries have often reported no significant gender differences in legal knowledge among healthcare professionals [25, 26]. The disparity observed in our study could be attributed to various factors specific to the Iraqi context, such as differences in educational opportunities, professional development pathways, or cultural influences on career choices and specialization within nursing. This finding highlights the importance of considering gender‐specific approaches to legal education and professional development in the Iraqi healthcare system. The strong association between positive attitudes toward legal issues and higher legal knowledge, coupled with the relationship between greater legal knowledge and lower interference in legal matters, underscores the interconnected nature of these factors. This interplay has been observed in studies from various healthcare settings, emphasizing the multifaceted impact of legal education on nursing practice [27, 28]. However, the specific manifestations of these relationships may differ in the Iraqi context due to unique cultural, legal, and healthcare system characteristics. Understanding these nuances is crucial for developing effective interventions that address the particular needs and challenges faced by emergency nurses in Erbil hospitals.

**Figure 1:**
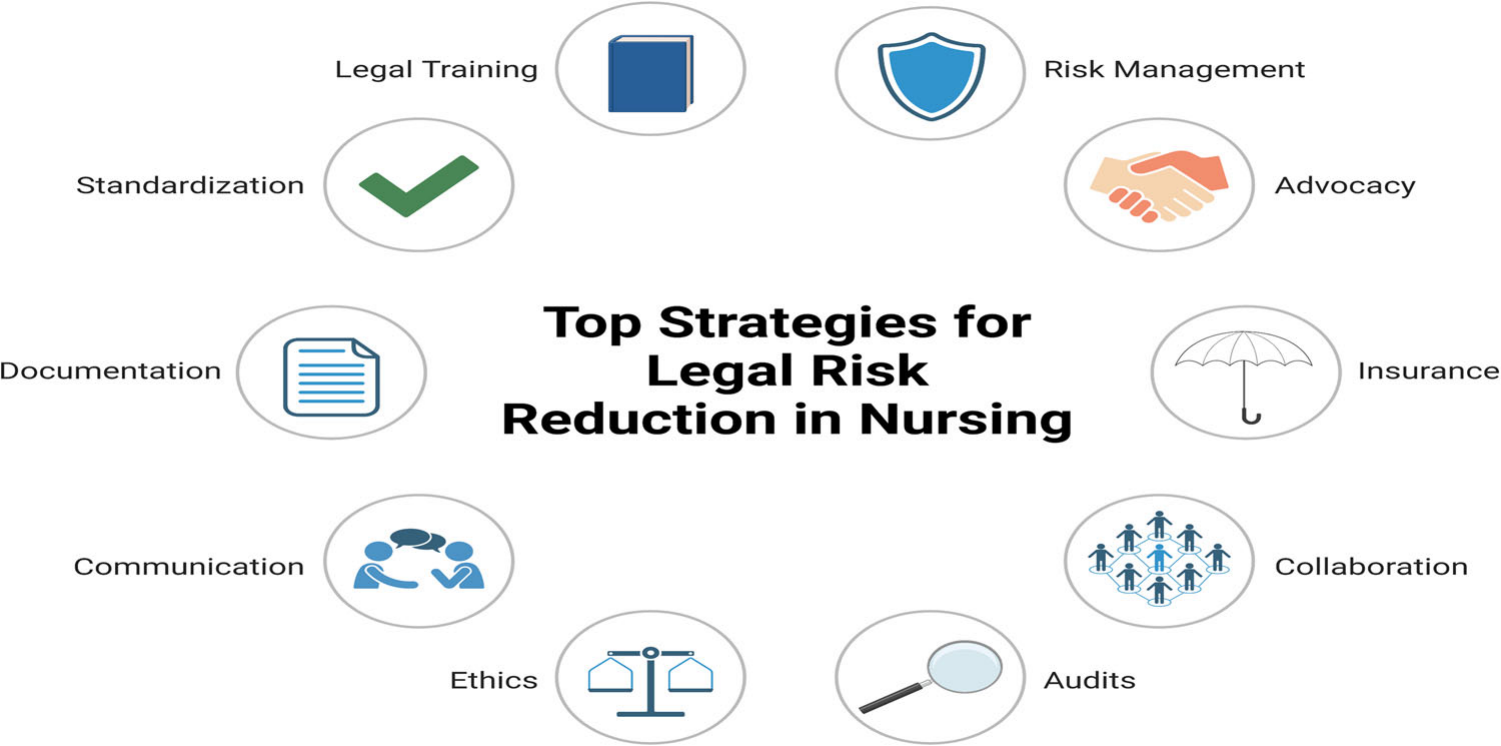
Top strategies for legal risk reduction in nursing. Created with BioRender.com.

There are several strategies that can be considered for nurses to reduce legal risks in their practice. These include ongoing legal education, robust documentation practices, and regular training on patient rights and consent procedures. Implementing these strategies can help nurses navigate legal complexities more effectively and enhance patient care. For further details, refer to Figure 1.

## 5 Conclusion

The study demonstrated that emergency nurses in Erbil have moderate levels of legal knowledge and attitudes toward legal issues, along with medium levels of interference in legal matters. This suggests that while there is a foundational understanding, significant improvements are necessary. Policymakers and healthcare providers should develop targeted educational interventions to enhance legal literacy among nurses. These programs should focus on key legal principles and practical applications to ensure nurses are well‐prepared to handle legal challenges. By improving legal knowledge, nurses can provide better patient care, reduce legal risks, and feel more confident in their professional roles. Enhanced legal literacy will ultimately lead to a safer and more effective healthcare environment.

## 6 Limitations and Directions for Future Research

A key limitation of the study is that its findings are specific to the eight major public hospitals in Erbil and may not be generalizable to other regions of Iraq or other populations of nurses. Future research should consider a longitudinal approach to better understand the causal relationships between legal knowledge, attitudes, and interference with legal issues among nurses. Furthermore, expanding the study to include nurses from different regions and healthcare settings in Iraq would provide a more comprehensive understanding of the legal awareness and needs of nurses across the country.

## Author Contributions

**Hardi Abdulqadir Hasan:** conceptualization, data curation, methodology, writing – original draft, visualization, writing – review and editing. **Abdulmalik Fareeq Saber:** conceptualization, methodology, software, data curation, formal analysis, project administration. **Alireza Nikbakht Nasrabadi:** conceptualization, formal analysis, investigation, methodology, project administration, supervision, writing – review and editing.

## Disclosures

This study is a part of the master's thesis in the Medical‐Surgical Nursing department at Tehran University of Medical Science.

## Ethics Statement

Ethical code was obtained from the School of Nursing and Midwifery & Rehabilitations—Tehran University of Medical Sciences (IR.TUMS.FNM.REC.1402.185), approved on 2023‐12‐16. Oral informed consent was obtained from all participants before they filled out the questionnaires. There are no reproduced materials in the current study.

## Consent

Oral informed consent was obtained from all participants before they filled out the questionnaires.

## Conflicts of Interest

The authors declare no conflicts of interest.

## Transparency Statement

The lead author Alireza Nikbakht Nasrabadi affirms that this manuscript is an honest, accurate, and transparent account of the study being reported; that no important aspects of the study have been omitted; and that any discrepancies from the study as planned (and, if relevant, registered) have been explained.

## Data Availability

The data that support the findings of this study are available from the corresponding author upon reasonable request.
